# Effect of Sucrose on the Formation of Advanced Glycation End-Products of Ground Pork during Freeze–Thaw Cycles and Subsequent Heat Treatment

**DOI:** 10.3390/foods12051024

**Published:** 2023-02-28

**Authors:** Fuyu Chu, Yi Lin, Yiqun Huang, Lihong Niu, Keqiang Lai

**Affiliations:** 1 College of Food Science and Technology, Shanghai Ocean University, No. 999 Hucheng Huan Road, LinGang New City, Shanghai 201306, China; 2 Engineering Research Center of Food Thermal-Processing Technology, Shanghai Ocean University, Shanghai 201306, China; 3 School of Food Science and Bioengineering, Changsha University of Science & Technology, 960, 2nd Section, Wanjiali South Road, Changsha 410004, China; 4 School of Food Engineering, Ludong University, No. 186, Middle Hongqi Road, Yantai 264025, China

**Keywords:** sucrose, freeze–thaw cycles, advanced glycation end-products, pork, correlation analysis

## Abstract

The changes in protein degradation (TCA-soluble peptides), Schiff bases, dicarbonyl compounds (glyoxal-GO, methylglyoxal-MGO) and two typical advanced glycation end-products (AGEs) including *N^ε^*-carboxymethyllysine (CML), *N^ε^*-carboxyethyllysine (CEL) levels in ground pork supplemented with sucrose (4.0%) were investigated under nine freeze–thaw cycles and subsequent heating (100 °C/30 min). It was found that increase in freeze–thaw cycles promoted protein degradation and oxidation. The addition of sucrose further promoted the production of TCA-soluble peptides, Schiff bases and CEL, but not significantly, ultimately leading to higher levels of TCA-soluble peptides, Schiff bases, GO, MGO, CML, and CEL in the ground pork with the addition of sucrose than in the blank groups by 4%, 9%, 214%, 180%, 3%, and 56%, respectively. Subsequent heating resulted in severe increase of Schiff bases but not TCA-soluble peptides. Contents of GO and MGO all decreased after heating, while contents of CML and CEL increased.

## 1. Introduction

Animal protein has an important role in people’s dietary life. Pork is a common source of animal protein intake in our daily life, and its muscle contains up to 20% protein. During the storage and transportation of pork products, proteins are prone to denaturation, oxidation, degradation and other adverse reactions, which eventually lead to product deterioration [1]. Refrigerated storage is generally considered to be an effective way to maintain food quality by slowing down the growth of spoilage microorganisms and inhibiting the activity of enzymes [1]. However, cold chain technology still has limitations and frozen meat will inevitably experience temperature fluctuations during transportation, storage and consumption, which will lead to the quality of fresh meat decreasing [2,3].

Fresh meat in the process of repeated freeze–thaws (FT) will not only promote lipid oxidation, protein degradation and other reactions, but also promote the accumulation of harmful substances such as Maillard end-products [3]. Du et al. [2] found that after multiple freeze–thaw cycles, the water holding capacity and thermal stability of carp decreased significantly, and oxidation reactions in the fish increased with the number of freeze–thaw cycles. After five FT, the water content, texture and color of yellow croaker changed significantly, and the increased protein oxidation reaction contributed to the deterioration of fish quality [4]. The deterioration of meat quality during freezing and thawing was due to the irreversible damage to muscle tissue and cells caused by ice crystals generated during freezing and recrystallization by repeated freezing and thawing [4,5].

Advanced glycation end-products (AGEs) are a group of chemically stable substances produced by complex reactions of reducing sugars and reactive carbonyl groups with amino groups of peptides, proteins, amino acids and nucleic acids under non-enzymatic conditions, and are generated by multiple pathways such as the Maillard reaction and lipid oxidation [6,7]. Among them, CML and CEL are often used as the basis for judging the content of AGEs in the body or food. The accumulation of AGEs in the human body is associated with chronic diseases such as diabetes, atherosclerosis, and Alzheimer’s disease [6]. The production of AGEs is influenced by various factors. Bosch et al. [8] studied the changes in AGEs content of milk-grain stored at three temperatures (25, 30 and 37 °C) for 9 months, and showed that AGEs increased with storage time, with higher temperatures leading to faster production. Kong et al. [9] found that high concentrations of NaCl inhibited the occurrence of protein degradation and lipid oxidation in dried mackerel treated with different salt concentrations for 25 days of storage (25 °C), which helped to control the formation of CML and CEL in the cured fish meat during storage. Li’s study [3] showed that freeze–thaw treatment accelerated the increase of AGEs in surimi products and subsequent heat-induced processes.

There are numerous studies on cryoprotectants, of which 4.0% sucrose, 4.0% sorbitol and 0.15% polyphosphate are often used as commercial cryoprotectants, also known as traditional cryoprotectants. Considering the cost and other issues, food products are most often stored, transported, and sold with 4.0% sucrose as a cryoprotectant to inhibit ice crystal damage to the food [10]. However, in recent years, people pay more and more attention to green and safe food; the addition of sucrose, on the one hand, will affect the taste and flavor of food, on the other hand, is not conducive to the consumption of patients such as diabetics. AGEs are a class of harmful substances that can cause chronic diseases such as diabetes and atherosclerosis, and the type of sugar also affects their production [6]. In fish cakes with sucrose added, both CML and fluorescent AGE increased, though not necessarily fluorescent, as Shi et al. found [11]. Although there are numerous studies on the application of sucrose as a cryoprotectant to foods, there are few reports on whether the addition of sucrose is harmful to freeze–thawed foods.

In this study, the protein degradation trend and the contents of Schiff bases, GO, MGO, CML and CEL of ground pork with 4.0% sucrose added as cryoprotectant were investigated after nine freeze–thaw cycles and subsequent heat treatment. The aim of the study was to investigate the effects of sucrose on AGEs through lipid oxidation, protein degradation and protein oxidation pathways; unlike other studies, we investigated the adverse effects of sucrose on meat products from the perspective of harmful food substances. The study intended to indicate the disadvantages of sucrose as a commercial cryoprotectant, to consider the appropriateness of adding sucrose to meat products, and to add new knowledge about sucrose.

## 2. Materials and Methods

### 2.1. Reagents

AGEs standards including CML, CEL, d4-CML and d4-CEL (≥98%) were purchased from Toronto Research Chemicals Inc. (Toronto, ON, Canada). HPLC grade methanol (99.9%), acetonitrile (99.9%), formic acid (98%), and LC-MS grade ammonium acetate (99%), 5-methylquinoxaline (5-MQ, 98%) were acquired from J&K Technology Co., LTD. (Beijing, China). GO (40.9%) and MGO (40%) were purchased from Chem Servive (West Chester, PA, USA) and Fluorochem (Hadfield, UK), respectively. All other reagents were of analytical grade and obtained from Sinopharm Chemical Reagent Co., Ltd. (Shanghai, China).

### 2.2. Sample Preparation

Fresh pork tenderloin was purchased from a local supermarket (Aisen Pork Food Co., Shanghai, China). The pig breed was a Duroc × Landrace × Large white cross, lean type, 8–9 months old. After transformed to the laboratory in a cooler, the visible connective and fatty tissues were cut off, the remaining meat was chopped and ground with a homogenizer (HGBTWTS3, Waring Commercial Corporation, Stamford, CT, USA). After each 10 s of mincing, the meat was cooled in an ice water bath for 7 s, and totally repeated for 6 times. Weighed 60.0 ± 0.1 g of churned pork, added 4.0% sucrose and stirred well, and labeled as sucrose groups. Ground pork without added sucrose were used as a blank control group (CK groups) [11]. The ground pork samples were sealed into PE bags separately. The CK groups and Suc groups were stored at −20 °C for 12 h, then thawed under 4 °C for 12 h, and thus cycled nine times. Two random patties of the same treatment were used for the follow-up measurements, respectively, at the 0th (0 FT), 3rd (3 FT), 6th (6 FT) and 9th (9 FT) freeze–thaw cycles. Totally 2 batches of ground pork were prepared. All indexes were measured in three parallel groups.

### 2.3. Heat Treatment

Sample of 13.0 ± 0.1 g from one pork patty was sealed in a cylindrical aluminum cell (diameter 50 mm; height 5 mm) [12] and heated at 100 °C for 30 min. Then, the cell was quickly immersed in ice-water mixture to cool down. The heated meat sample along with the exuded juice were collected, ground and mixed with a mortar, and then sealed in a plastic bag before further use.

### 2.4. Determination of Trichloroacetic Acid (TCA)-Soluble Peptides

According to Lin et al. [13], 27 mL of pre-cooled 5% TCA solution was added into 3.0 g of pork sample. The mixture was homogenized for 1 min and then let stand in an ice water bath for 1 h. Following this, the mixture was centrifuged at 4 °C for 10 min (8000× *g*) and the TCA-soluble peptides content in the supernatant was determined with Lowry’s method [14]. Results were calculated as μmol Tyrosine/g sample.

### 2.5. Detection of Schiff bases

The content of Schiff bases was determined using the method of Renerre, Dumont and Gatellier [15] with slight modifications. Approximately 0.5 g of raw pork was mixed with 2.5 mL of 20 mmol/L phosphate buffer (containing 100 mmol/L NaCl, pH 6.0), and homogenized for 30 s. The homogenate of 1 mL was mixed with 4 mL of dichloromethane: ethanol (2:1, *v*/*v*), and centrifuged for 15 min (4000× *g*) at 4 °C. The fluorescence intensity of the supernatant was detected using a fluorescence spectrophotometer (RF-5301PC, Shimadzu Corporation, Kyoto, Japan) with excitation wavelength of 327 nm, emission wavelength of 380–600 nm, and emission slit of 5.0 nm. 

### 2.6. Extraction and Detection of GO and MGO

The levels of two di-carbonyls, GO and MGO, were measured following the method of Li et al. [16]. Pork sample of 1.0 g was added into 10 mL of 0.6 mol/L perchloric acid with 5-MQ as internal standard. After homogenization, the mixture was placed at 4 °C for 15 min, and then centrifuged at 4 °C for 10 min (8000× *g*), and 1 mL of the supernatant was added with 0.2 mL of 5 mg/mL o-phenylenediamine and reacted at 60 °C for 3 h in water bath. After cooling to room temperature, the sample was purified with a 0.22 μm PTFE filter and used for detection. 

The above extract was analyzed with HPLC (Agilent 1260, Agilent Inc., Santa Clara, CA, USA). A Capcell PAK C18 AQ (4.6 mm I.D. × 250 mm, Agilent) column was used, and the column temperature was set at 25 °C with an injection volume of 20 μL. Mobile phases were A: 0.15% (*v*/*v*) acetic acid solution and B: acetonitrile. Gradient elution was set as following based on mobile phase B: 0–10 min, 8–40%; 10–12 min, 40–48%; 12–13 min, 48–60%; 13–15.5 min, 60–80%; 15.5–20.5 min, 80–8%. The flow rate was 0.8 mL/min and the detection wavelength was 313 nm.

### 2.7. Extraction and Detection of CML and CEL

The extraction and detection of protein-bound CML and CEL of pork samples were based upon a HPLC-MS/MS method previously explored by Sun, Tang, Wang, Rasco, Lai, & Huang [17] and Niu et al. [18]. Briefly, 0.21 g of pork sample was mixed with 2 mL of 0.2 mol/L borate buffer and 0.4 mL of 2 mol/L sodium borohydride, mixed and reduction at 4 °C for 8 h. Subsequently, 4 mL of chloroform-methanol solution (*v*/*v* = 2:1) was added, mixed, and centrifuged at 4 °C for 10 min (5000× *g*) to remove fat and precipitate protein. The precipitated protein was hydrolyzed at 110 °C for 24 h. Then, the hydrolysate was filled to 10 mL, and 4 mL was spiked with internal standards (d4-CML and d4-CEL) and vacuum-dried at 50 °C. The dried sample was reconstructed with 3 mL ultrapure water and cleaned with a MCX column (60 mg/3 mL, Shanghai Ampli Experimental Co. Ltd., Shanghai, China). The eluent was dried under nitrogen at 50 °C and dissolved in 2 mL methanol-water solution (80:20, *v*/*v*). Sample was finally purified with 0.22 μm PTFE filter before HPLC-MS/MS analysis. 

An Altus-30 ultra-performance liquid chromatography (UPLC) system (Perkin Elermer Inc., manufactured in Shelton, Shelton, CT, USA) with an Atlantis HILIC column (150 × 2.1 mm, 3 μm, Waters Corp. Milford, MA, USA) and a Waters Quattro Micro triple quadrupole tandem mass spectrometry system (AB Sciex Pte. Ltd., Marsiling, Singapore) were used for detection of CML and CEL, following the method of Li [3].

### 2.8. Data Analysis

The number of freeze–thaw and heating treatments were used as independent variables and TCA-soluble peptides, Schiff bases, GO, MGO, CML and CEL as dependent variables. Analysis of variance (ANOVA) was performed using SPSS 22.0 software (IBM, Chicago, IL, USA) to assess whether there were significant differences (α = 0.05 level) in the mean levels of the dependent variable indicators in pork during freeze–thaw and subsequent heating. Data were expressed as mean ± standard deviation.

## 3. Results and Discussion

### 3.1. Changes of TCA-Soluble Peptides in Ground Pork during Freeze–Thaw Cycles and Subsequent Heating 

Table 1 shows the changes in the content of TCA-soluble peptides in ground pork during freeze–thaw treatment and subsequent heating. The content of TCA-soluble peptides increased significantly in both the CK and Suc groups after nine freeze–thaws, from 1.73 ± 0.03 (0 FT) to 2.11 ± 0.01 (9 FT) in the CK groups and from 1.93 ± 0.02 (0 FT) to 2.13 ± 0.04 (9 FT) in the Suc groups for raw meat. The increase in the content of TCA-soluble peptides indicated that the proteins were continuously degraded by proteases and microorganisms, that the ice crystals formed during the freezing treatment can break the structure of muscle cells, and that the exudation of intracellular solutes after thawing promoted the growth of microorganisms and the production of more hydrolyzed peptides [3,19].

The initial value of the fresh meat Suc groups (1.93 ± 0.02, 0 FT) was higher than that of the CK groups (1.73 ± 0.03, 0 FT). Since the degradation of protein was a combination of proteases and microorganisms, we speculated that the added sucrose may be degraded by the endocrine invertase of sucrose. It provided more carbon substrates and energy for the growth of microorganisms [20], thus promoting the degradation of proteins. However, TCA-soluble peptides content was increased by 22% in CK groups but 10% in Suc groups after 9 FT. The growth rate of the Suc groups was significantly smaller than that of the CK groups, and we speculated that the free hydroxyl groups in sucrose bound to the water molecules of the Suc groups during the subsequent freeze–thaw process, inhibiting the cell destruction caused by ice crystal formation, thereby slowing down the degradation of proteins by microbial growth [21].

After heat treatment at 100 °C/30 min, a small increase (<10%) of TCA soluble peptide was observed in both CK and Suc groups. This is despite the fact that heat treatment has been shown to promote protein degradation in tuna whitebait (45 °C for 30 min and 90 °C for 20 min) and abalone (60, 70, 80 and 100 °C for 10 min) [3,22]. Most endogenous proteases in muscle were inactivated at 70 °C. The peptide content at 100 °C was mainly influenced by cross-linking of small molecule peptides and degradation of high molecule peptides [23].

### 3.2. Changes of Schiff Bases in Ground Pork during Freeze–Thaw Cycles and Subsequent Heating

Schiff bases are a group of unstable compounds containing C = N bonds that can be produced by the reaction of the free amino groups of lysine, arginine and glutamine residues in proteins with the aldehyde groups in reducing sugars in the early stages of the Maillard reaction [6]. The level of Schiff bases is often used to reflect the oxidation of proteins [3].

No significant effect of freeze–thaw treatment on Schiff bases was found in our study (Table 2), which may be due to the dynamic changes in the rate of Schiff bases formation and degradation due to further oxidation and destruction of the Schiff bases as an intermediate product of the unstable Maillard reaction while generating carbonyl compounds or more stable Amadori products [3,16]. However, in raw meat, the mean fluorescence intensity of Suc groups (140.98 a.u) was higher than that of CK groups (129.19 a.u), which might be due to the reduction sugar produced by the degradation of sugars caused by the catalyst in the cytoplasm, which would promote the Maillard reaction [24].

Heat treatment resulted in a highly increased fluorescence intensity, which increased with the number of freeze–thaw cycles by a factor of 225% (CK groups) and 160% (Suc groups), respectively (Table 2). High temperature may promote the Maillard reaction, leading to significant oxidation of proteins [25]. However, after heat treatment, the mean fluorescence intensity in the Suc groups (366.75 a.u.) was lower than that in the CK groups (418.78 a.u.); the reason for this result remains unclear and requires further investigation.

### 3.3. Changes of Dicarbonyl Compounds (GO, MGO) in Ground Pork during Freeze–Thaw Cycles and Subsequent Heating

**Figure 1:**
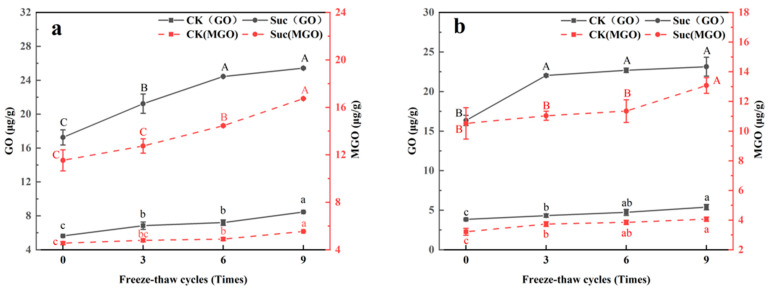
Changes of glyoxal (GO) and methylglyoxal (MGO) contents in ground pork during freeze–thaw cycles and subsequent heating. (**a**). raw; (**b**). 100 °C, 30 min cooked). ^a,b,c^ Different letters indicate significant difference (α = 0.05) of GO and MGO contents in CK groups during freeze–thaw cycles. ^A,B,C^ Different letters indicate significant difference (α = 0.05) of GO and MGO contents in Suc groups during freeze–thaw cycles.

As shown in Figure 1a, after nine freeze–thaws, the content of GO and MGO in both the CK (50% and 22%) and Suc (47% and 45%) groups of fresh meat increased significantly. This may be due to the fact that the freeze–thaw process, while destroying cells, led to the release of pro-oxidants (lysosomal enzymes, heme iron) in the cytosol [26], which promoted lipid oxidation and accelerated the production of GO and MGO.

The GO content and MGO content in the Suc groups were about 214% and 180% higher than those in the CK groups for fresh meat. This was caused by the degradation of sucrose in the Suc groups during storage and freeze–thaws, which degraded to free glucose and fructose [24]. On the one hand, 1-deoxyglucosone (1-DG) and 3-deoxyglucosone (3-DG) generated from glucose and Heyns products (formed by the reaction of fructose with amines) by 1,2-enolization undergo further inverse hydroxyl aldol condensation to form MGO; on the other hand, the glucuronide produced by oxidation of glucose via the 1,2-enediol pathway is further degraded to produce GO [24,27]. In the raw ground pork added sucrose, the degradation reaction of sugar was more intense and, therefore, led to the production of more GO and MGO.

Heat treatment (100 °C, 30 min) resulted in a significant reduction of GO and MGO content in the ground pork (Figure 1b). Due to heating, the average levels of GO decreased by 35% (CK groups) and 5% (Suc groups), respectively, while MGO decreased by 25% (CK groups) and 17% (Suc groups). The results were consistent with the study by Li et al. [16] which reported a 16% and 13% reduction in GO and MGO, respectively, in commercially sterilized pork (121 °C, 10 min). This may be due to the fact that the production and conversion of GO and MGO occurred simultaneously and the rate of their production was limited by the initial concentration of reducing sugars [28,29]. Meanwhile, dicarbonyl compounds can be trapped by various amino acids and the reaction rate depended on the amino acid content, while heating would promote the trapping ability of amino acids [28,30]. Therefore, it was conjectured that the reducing sugars of ground pork were continuously consumed during the heating process, while the amino acids generated from reactions such as protein degradation continuously combine with dicarbonyl compounds for further reactions, resulting in a reaction rate of GO and MGO greater than the production rate, which eventually caused a decrease in the content of GO and MGO. In addition, the decrease in the Suc groups after heat treatment was less than that in the CK groups, which was due to the degradation of sucrose in the Suc groups that promoted the production of GO and MGO [24,27].

### 3.4. Changes of CML and CEL in Ground Pork during Freeze–Thaw Cycles and Subsequent Heating

**Figure 2:**
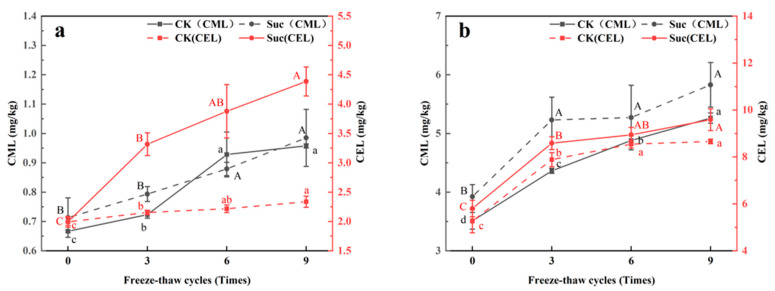
Changes of *N^ε^*-carboxymethyllysine (CML) and *N^ε^*-carboxyethyllysine (CEL) contents in ground pork during freeze–thaw cycles and subsequent heating. (**a**). raw; (**b**). 100 °C, 30 min cooked). ^a,b,c,d^ Different letters indicate significant difference (α = 0.05) of CML and CEL contents in CK groups during freeze–thaw cycles. ^A,B,C^ Different letters indicate significant difference (α = 0.05) of CML and CEL contents in Suc groups during freeze–thaw cycles.

Figure 2 shows the changes in CML and CEL contents of ground pork in the CK and Suc groups under nine freeze–thaws and during subsequent heating. As shown in Figure 2a, the CML increased from 0.67 ± 0.01 (0 FT) to 1.16 ± 0.04 mg/kg (9 FT) and the CEL increased from 1.99 ± 0.09 (0 FT) to 2.24 ± 0.10 mg/kg (9 FT) in the CK groups and from 0.71 ± 0.07 (0 FT) to 0.89 ± 0.02 mg/kg (9 FT) in the Suc groups after freeze–thawing fresh meat; the CML increased from 0.71 ± 0.07 (0 FT) to 0.89 ± 0.02 mg/kg (9 FT), and CEL increased from 1.99 ± 0.06 (0 FT) to 4.39 ± 0.25 mg/kg (9 FT). According to Li et al. [3], the fluorescent AGEs of surimi products in the freeze–thaw cycles were increased by 21.84% (3 FT) and 44.96% (6 FT), respectively, compared to fresh samples. Wu et al. [31] showed that after seven freeze–thaw cycles, the average levels of CML and CEL were 14.02% and 11.81% higher than those of fresh pork, respectively. This was due to the fact that the freeze–thaw treatment accelerated the degradation of the protein to form small molecular peptides (Table 1), exposing more amino groups involved in the Maillard reaction. On the other hand, the freeze–thaws promoted the oxidation reaction and the generated GO and MGO (Figure 1) further reacted with lysine to form CML and CEL.

In the fresh ground pork, the average levels of CML (0.82 mg/kg) and CEL (2.18 mg/kg) in the CK groups were lower than those of CML (0.84 mg/kg) and CEL (3.39 mg/kg) in the Suc groups. On the one hand we found a higher degree of protein degradation in the Suc groups (Table 1), which would provide more amino and amino acids involved in the reaction to generate AGEs. On the other hand, sugar degradation was higher in the Suc groups, and reducing sugars would facilitate the Maillard reaction [6,32]. In addition, GO and MGO, produced with the participation of reducing sugars, would combine with lysine to further react to produce CML and CEL [24,27].

After heat treatment at 100 °C for 30 min, the levels of both CML and CEL increased significantly (Figure 2b). After subsequent heating, the average levels of CML increased by 450% and 501% and CEL increased by 249% and 142% in the CK and Suc groups, respectively. Similar results were reported by Sun et al. [17] and Niu et al. [25] that heat treatment could significantly promote the formation of CML and CEL in muscle.

### 3.5. Correlation Analysis between Freeze–Thaws (FT), TCA-Soluble Peptides, Schiff Bases, GO, MGO, CML and CEL Indicators

To understand the effect of multiple freeze–thaw cycles on the formation of AGEs in pork, we correlated the levels of FT, TCA-soluble peptides, Schiff bases, dicarbonyl compounds (GO, MGO) and AGEs (CML, CEL) in ground pork (both raw and heated) in the CK and Suc groups. As shown in Table 3, the levels of CML and CEL were significantly and positively correlated with FT, TCA-soluble peptides and GO, but not with Schiff bases in raw ground pork. The results were consistent with the above finding that the levels of Schiff bases in raw pork did not change significantly during freeze–thaws, but the levels of both CML and CEL increased. The levels of both CML and CEL were significantly correlated with MGO in the Suc groups (r = 0.944 and 0.876), but not in the CK groups. This may be due to the fact that the addition of sucrose further promotes protein degradation and oxidation reactions, which lead to the production of AGEs.

In cooked meat (Table 4), the correlation of Schiff bases with both CML and CEL increased (r = 0.846 and 0.887, CK groups; r = 0.844 and 0.622, Suc groups). Based on our above findings, the effect of freeze–thaws on Schiff bases was not significant, while the level of Schiff bases showed an increasing trend with increasing number of freeze–thaws during the subsequent heating. This may be due to the accumulation of Schiff bases precursors, such as aldehydes from lipid oxidation and peptides from protein degradation, in pork products after freeze–thaw cycles, which may contribute to the formation of Schiff bases at higher temperatures.

## 4. Conclusions

Based on the above results, we hypothesized. The microbial growth and the release of oxidation promoters (lysosomal enzymes, heme iron, etc.) caused by freezing and thawing would act coordinately on the protein degradation and oxidation reactions, ultimately promoting the accumulation of AGEs through the Maillard reaction and lipid oxidation pathways. In contrast, the correlation between GO, MGO and AGEs became less significant due to the fact that sucrose significantly promotes the accumulation of GO and MGO through sugar degradation (Figure 1), while GO and MGO may be involved in other reactions and only a small fraction of them bind to lysine to generate AGEs. Therefore, compared to the CK groups, CML and CEL were less increased in the Suc groups (Figure 2), which ultimately led to the correlation between dicarbonyl compounds (GO, MGO) and AGEs (CML, CEL) was decreased.

Whereas the addition of sucrose would not significantly promote protein degradation and protein oxidation, probably due to the fact that sucrose provided a carbon base and energy for microbial growth while binding free water, thereby inhibiting cell damage by ice crystal formation; whereas the glucose generated from sucrose via the sugar degradation pathway and the Heyns product through 1,2-enolization produced 1-deoxyglucosone (1-DG) and 3-deoxyglucosone (3-DG) undergo further inverse hydroxylaldehyde condensation to produce MGO; on the other hand, further degradation of glucuronides generated by oxidation of glucose via the 1,2-enediol pathway generates GO, which eventually also contributed to the accumulation of AGEs.

The contents of TCA-soluble peptides, GO, MGO, CML and CEL in ground pork from both CK and Suc groups increased with the number of freeze–thaws, indicating that protein degradation and lipid oxidation reactions occurred continuously during freeze–thaws, further promoting the production of AGEs. In contrast, freeze–thaw treatments did not have a significant effect on Schiff bases. The different effects of heat treatment on TCA-soluble peptides, Schiff bases, GO, MGO and CML, and CEL showed that heating had no significant effect on protein degradation, but accelerated AGE production by promoting the Maillard reaction and oxidation pathway.

The results for TCA-soluble peptides and Schiff bases in raw meat suggested that the addition of sucrose as an cryoprotectant agent may promote further degradation of sucrose to more reducing sugars by catalysts in the cytoplasm, facilitating the Maillard and oxidation reactions and ultimately leading to more AGEs generation. However, this facilitation response was not significant, probably because sucrose, while degrading, also bound to free water thus limiting the damage to cells by ice crystal production. Therefore, the promotion and inhibition of protein degradation and oxidation by the addition of sucrose may coexist. However, sucrose significantly promoted GO and MGO levels through the sugar degradation pathway, which ultimately also promoted the accumulation of AGEs (more significantly for CEL). Based on our study on the content of AGEs in pork with added sucrose, we showed that the addition of sucrose further promotes the accumulation of AGEs in food. Therefore, a greener, more efficient and safer cryoprotectant should be chosen instead of sucrose in the food storage and transportation process. Our research should also focus more on the development and application of new cryoprotectants.

## Figures and Tables

**Table 1:** Changes of TCA-soluble peptides contents in ground pork during freeze–thaw cycles and subsequent heating (100 °C, 30 min).

Group	Freeze–Thaw Cycle/Times	TCA-Soluble Peptides (μmol/g)	
		Raw	Cooked
CK	0	1.73 ± 0.03 ^d^	1.92 ± 0.05 ^c^
	3	1.93 ± 0.01 ^c^	2.11 ± 0.01 ^b^
	6	1.99 ± 0.01 ^b^	2.15 ± 0.03 ^ab^
	9	2.11 ± 0.01 ^a^	2.23 ± 0.02 ^a^
Suc	0	1.93 ±0.02 ^C^	2.02 ± 0.02 ^C^
	3	2.00 ± 0.02 ^BC^	2.05 ± 0.02 ^C^
	6	2.03 ± 0.02 ^B^	2.11 ± 0.01 ^B^
	9	2.13 ± 0.04 ^A^	2.25 ± 0.01 ^A^

^a,b,c,d^ Different letters indicate significant difference (α = 0.05) of TCA-soluble peptides contents in CK groups during freeze–thaw cycles; ^A,B,C^ Different letters indicate significant difference (α = 0.05) of TCA-soluble peptides contents in Suc groups during freeze–thaw cycles.

**Table 2:** Changes of Schiff bases contents in ground pork during freeze–thaw cycles and subsequent heating (100 °C, 30 min).

Group	Freeze–Thaw Cycle/Times	Fluorescence (a.u.)	
		Raw	Cooked
CK	0	122.86 ± 11.06 ^a^	368.97 ± 21.92 ^c^
	3	130.26 ± 3.16 ^a^	411.10 ± 20.42 ^bc^
	6	131.97 ± 3.80 ^b^	441.66 ± 18.69 ^b^
	9	131.68 ± 8.53 ^a^	453.41 ± 25.77 ^a^
Suc	0	140.91 ± 9.07 ^A^	335.26 ± 20.50 ^B^
	3	140.79 ± 13.55 ^A^	358.97 ± 17.79 ^AB^
	6	141.44 ± 0.70 ^A^	387.22 ± 18.86 ^A^
	9	141.03 ± 12.68 ^A^	385.53 ± 8.42 ^A^

^a,b,c^ Different letters indicate significant difference (α = 0.05) of Schiff bases contents in CK during freeze–thaw cycles. ^A,B^ Different letters indicate significant difference (α = 0.05) of Schiff bases contents in Suc groups during freeze–thaw cycles.

**Table 3:** Correlation analysis between freeze–thaws (FT), TCA-soluble peptides, Schiff bases, GO, MGO, CML and CEL indicators in raw meat.

Group	Index	FT	TCA- Soluble Peptides	Schiff Bases	GO	MGO	CML	CEL
CK								
	FT	1						
	TCA-soluble peptides	0.970 **	1					
	Schiff bases	0.491	0.484	1				
	GO	0.968 **	0.947 **	0.480	1			
	MGO	0.736 *	0.670	0.610	0.767 *	1		
	CML	0.934 **	0.869 **	0.403	0.882 **	0.534	1	
	CEL	0.776 *	0.803 *	0.424	0.824 *	0.407	0.762 *	1
Suc								
	FT	1						
	TCA-soluble peptides	0.952 **	1					
	Schiff bases	0.015	−0.124	1				
	GO	0.959 **	0.893 **	−0.113	1			
	MGO	0.972 **	0.929 **	−0.123	0.929 **	1		
	CML	0.916 **	0.799 *	0.054	0.892 **	0.944 **	1	
	CEL	0.947 **	0.926 **	0.022	0.954 **	0.876 **	0.802 *	1

Note: * and ** represent a significant correlation at 0.05 level and 0.01 level (two-tail test), respectively.

**Table 4:** Correlation analysis between freeze–thaws (FT), TCA-soluble peptides, Schiff bases, GO, MGO, CML and CEL indicators in cooked meat.

Group	Index	FT	TCA- Soluble Peptides	Schiff Bases	GO	MGO	CML	CEL
CK								
	FT	1						
	TCA-soluble peptides	0.942 **	1					
	Schiff bases	0.879 **	0.867 **	1				
	GO	0.851 **	0.774 *	0.837 **	1			
	MGO	0.751 *	0.788 *	0.651	0.828 *	1		
	CML	0.882 **	0.941 **	0.846 **	0.746 *	0.751 *	1	
	CEL	0.869 **	0.924 **	0.887 **	0.698	0.727 *	0.968 **	1
Suc								
	FT	1						
	TCA-soluble peptides	0.942 **	1					
	Schiff bases	0.816 *	0.693	1				
	GO	0.843 **	0.685	0.837 **	1			
	MGO	0.821 *	0.922 **	0.737 *	0.654	1		
	CML	0.830 *	0.729 *	0.844 **	0.860 **	0.726 *	1	
	CEL	0.783 *	0.718 *	0.622	0.859 **	0.691	0.702	1

Note: * and ** represent a significant correlation at 0.05 level and 0.01 level (two-tail test), respectively.

## Data Availability

The data used to support the findings of this study can be made available by the corresponding author upon request.
